# Global and regional dispersal patterns of hepatitis B virus genotype E from and in Africa: A full-genome molecular analysis

**DOI:** 10.1371/journal.pone.0240375

**Published:** 2020-10-08

**Authors:** Luicer Anne Olubayo Ingasia, Evangelia Georgia Kostaki, Dimitrios Paraskevis, Anna Kramvis

**Affiliations:** 1 Hepatitis Virus Diversity Research Unit, Department of Internal Medicine, School of Clinical Medicine, Faculty of Health Sciences, University of the Witwatersrand, Johannesburg, South Africa; 2 Department of Hygiene, Epidemiology and Medical Statistics, Medical School, National and Kapodistrian University of Athens, Athens, Greece; Centers for Disease Control and Prevention, UNITED STATES

## Abstract

Description of the spatial characteristics of viral dispersal is important in understanding the history of infections. Nine hepatitis B virus (HBV) genotypes (A-I), and a putative 10^th^ genotype (J), with distinct geographical distribution, are recognized. In sub-Saharan Africa (sub)-genotypes A1, D3 and E circulate, with E predominating in western Africa (WA), where HBV is hyperendemic. The low genetic diversity of genotype E (HBV/E) suggests its recent emergence. Our aim was to study the dispersal of HBV/E using full-length, non-redundant and non-recombinant sequences available in public databases. HBV/E was confirmed, and the phylogeny reconstruction performed using maximum likelihood (ML) with bootstrapping. Phylogeographic analysis was conducted by reconstruction of ancestral states using the criterion of parsimony on the estimated ML phylogeny. 46.5% of HBV/E sequences were found within monophyletic clusters. Country-wise analysis revealed the existence of 50 regional clusters. Sequences from WA were located close to the root of the tree, indicating this region as the most probable origin of the HBV/E epidemic and expanded to other geographical regions, within and outside of Africa. A localized dispersal was observed with sequences from Nigeria and Guinea as compared to other WA countries. Based on the sequences available in the databases, the phylogenetic results suggest that European strains originated primarily from WA whereas a majority of American strains originated in Western Central Africa. The differences in regional dispersal patterns of HBV/E suggest limited cross-border transmissions because of restricted population movements.

## Introduction

Hepatitis B virus (HBV) is a common cause of liver disease and the prototype member of the family *Hepadnaviridae* [1]. Despite the availability of an effective vaccine, HBV infections continue to be a public health problem [2, 3]. In 2015, the World Health Organization (WHO) estimated that over 257 million people are chronically infected with HBV [4–6]. Globally, HBV infections account for an estimated 887,000 deaths mostly from cirrhosis and hepatocellular carcinoma [4–8], with at least 250,000 of these recorded in Africa [7–9]. National and regional prevalence of HBV ranges from over 6% in Western Pacific and Africa, [5, 6, 10] with West Africa being the most affected, to under 0.7% in the United States and Northern Europe [5, 6, 10].

The unusual mechanism of HBV replication by reverse transcription and the lack of proof-reading ability of an RNA intermediate result in sequence heterogeneity [11, 12]. HBV is classified into at least 9 genotypes; A to I and a putative tenth genotype (J). Genotypes A–D, F, H and I are further classified into at least 35 subgenotypes [13].

Most HBV genotypes and subgenotypes have a distinct geographical distribution [13–15]. In sub-Saharan Africa, (comprising Eastern Africa, Central Africa, Southern Africa and Western Africa), HBV genotypes A, D and E circulate, with genotype A predominating in southern and eastern parts of the continent, while genotype D is found in the northern regions [16]. West Africa is the only major region in the world where HBV is still hyperendemic—[> 8% of hepatitis B surface antigen (HBsAg) chronic carriers]. HBV/E, which was first described in 1992 [15, 17] is the predominant genotype prevailing in this region [18].

The prevalence of HBV/E decreases in proportions towards Eastern Africa, where, with the exception of Madagascar (genotype E), mainly genotype A has been found [19]. HBV/E is rarely found outside Africa except in individuals of African descent [20], with sporadic cases reported in the Americas, [20–22] Northern Europe [23], including Belgium [24] and the Netherlands [25]. Despite the wide geographical distribution and dominance in sub-Saharan Africa, HBV/E has a very low genetic diversity ranging between 1.2% and 1.75% [16, 18, 26, 27]. This has led to the suggestion that this genotype was recently introduced into the human population ~300–6,000 years ago [28, 29] though, its high prevalence throughout the genotype E crescent is difficult to comprehend. HBV genotype A was the initial ancestral genotype in West Africa, which, in some areas, co-circulates with HBV/E [30, 31].

Previous studies have shown that the heterogeneity in the global distribution of the HBV genotypes may be responsible for the differences in the natural history of chronic HBV infections, clinical consequences, as well as the response to antiviral treatment [15, 16, 32, 33]. HBV/E has clinically been characterized, with significantly high viral loads and patients infected with this genotype are more likely to be hepatitis B e antigen (HBeAg)-positive than the patients infected with genotype D [14, 34, 35]. A higher HBeAg-positivity of this genotype has been shown to confer tolerance with a milder clinical manifestation [36, 37]. In addition, infection with HBV/E has previously been linked to higher chronicity rates than other genotypes [14, 34, 35].

Although significant differences in the patterns of dispersal of genotypes D and A have been shown, [29, 38] the dispersal patterns of the predominant genotype in West and Central Africa, HBV/E, is yet to be unraveled. The main aim of this study was to use all the available full-length sequences of this genotype, to estimate the levels of its regional dispersal and to shed light on geographical dissemination of genotype E.

## Materials and methods

### DNA sequence alignment, genotyping and recombination analysis

A total of 636 full-length sequences of HBV/E available in the public repositories; NCBI (http://www.ncbi.nlm.nih.gov) [39] and the Hepatitis B virus database (HBVdb; https://hbvdb.lyon.inserm.fr/HBVdb/) [40] were downloaded, including the geographic area of sampling. It should be noted that all genotype E sequences sampled from Europe and Americas may have been derived from HBV carriers of African origin regardless of their country of residence since genotype E is rarely found outside Africa. All sequences present in the databases as of August 2020 have been accessed. Duplicate sequences (N = 253) from the two public repositories identified by their identical accession number were removed from the analyses in addition to the sequences lacking the metadata (N = 42). Simplot v3.5 [41, 42] and RDP4 v4.36 [43] programs were used to detect the possible recombinant forms of the virus [42] and removed from the downstream analysis. Of the 636 downloaded sequences, 318 full-length non-recombinant and non-redundant sequences of HBV/E were used in the analyses.

The alignment of the full-length HBV/E sequences to representative complete genome sequences of the nine HBV genotypes A to J was performed by MUSCLE algorithm as implemented in MEGA v10 [44]. HBV genotyping was performed by the Oxford HBV Automated Subtyping Tool v1.0 [45]. Conserved signature motifs in the PreS1 [Leu^3^SerTrpThrValProLeuGluTrp^11^, His^15^, Thr^18^, Arg^38^, His^44^, Thr^52^, Met^83^, Lys^85^ and Thr^108^], specific for genotype E were confirmed [18]. In addition, all genotype E sequences had Arg^122^, Lys^160^ and Leu^127^ amino acid residues within the S gene and a Met^164^ amino acid substitution in the reverse transcriptase [18]. Furthermore, the spacer region contained eight amino acids residues unique to the genotype E: Met^64^, Glu^16^, His^21^, Arg^52^, Asp^55^, Lys^88^, Asn^110^ and His^111^. In order to determine diversity in the sequences from diverse geographical regions, nucleotide sequence divergence was performed on the complete nucleotide sequences using the divergence tool described by Bell and colleagues [46, 47].

### Country grouping, phylogenetic and phylogeographic analysis

**Fig 1:**
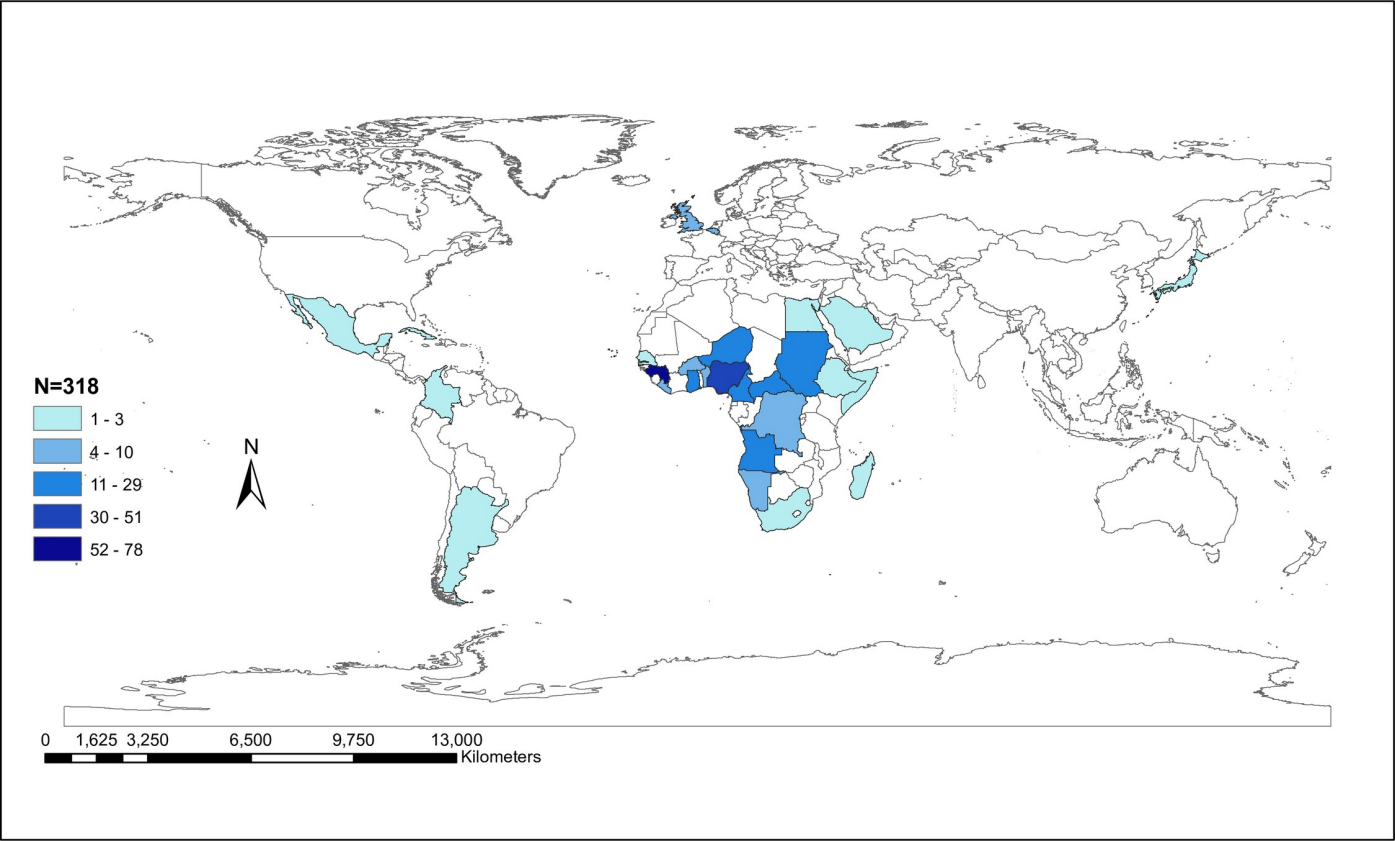
Global distribution of HBV/E sequences. Global map representing the distribution of available HBV/E full-length sequences by country of sampling for 29 countries around the world. Map was plotted using ArcGIS® software version 10.5 [49].

The available HBV/E sequences from different countries (N = 318) were classified into geographical regions according to the Global Burden of Disease classification system (http://www.who.int) [48]. The global distribution of these sequences per country as shown in Fig 1 was plotted using ArcGIS® software version 10.5 [49]. Phylogenetic analysis with bootstrap evaluation was performed using the maximum likelihood method with the Generalized Time Reversible (GTR+G) model of nucleotide substitution as implemented in RAxML v8.0.20 [50]. Monophyletic clusters were defined as those having bootstrap values higher than 70%, within which 70% of HBV/E strains share the same geographic area of sampling (country or region). Trees were converted to midpoint rooted by using the FigTree v1.4.3 program (http://tree.bio.ed.ac.uk/software/figtree/) [51]. The origin of genotypes E was inferred by character reconstruction using the criterion of parsimony on the estimated ML phylogeny using Mesquite v3.2 [52]. We conducted two kinds of phylogeographic analyses: one grouping sequences according to the country of sampling and another, grouping them according to the geographic regions as defined by the Global Burden of Disease classification system [48].

## Results

We studied 318 complete genome sequences sampled from 29 countries around the world, which showed a mean nucleotide diversity of 1.95% ranging between 0% and 3% (S1A Table). Nearly 93% of all sampled sequences were collected in Africa. Specifically, 54.5% of the HBV sequences were isolated from four African countries, namely Guinea (24.5%), Nigeria (16%), Cameroon (9.1%) and Central African Republic (9.1%) (Table 1, Fig 1). However, the highest mean nucleotide diversity of ~3% was observed for sequences sampled from United Kingdom and Belgium (S1B Table). In addition, the highest intergroup sequence divergence of ~3% between the countries was observed for Central African Republic and United Kingdom, Central African Republic and Belgium and United Kingdom and Belgium (S1C Table).

**Table 1:** Sampling of HBV/E sequences from different countries and percentages of clustering.

Country of Sampling	Number of sequences	Number of clustered sequences	Number of monophyletic clusters	Percentage of clustering (%)
Angola	18	-	-	-
Argentina	2	-	-	-
Belgium	6	2	1	33.3
Benin	4	-	-	-
Burkina Faso	10	-	-	-
Cameroon	29	23	4	79.3
Cape Verde	7	-	-	-
Central African Republic	29	14	6	48.3
Colombia	2	2	1	100.0
Cuba	2	-	-	-
Democratic Republic of the Congo	4	4	1	100.0
Egypt	2	2	1	100.0
Ethiopia	1	-	-	-
Ghana	15	9	4	60.0
Guinea	78	37	14	47.4
Japan	1	-	-	-
Liberia	6	4	2	66.7
Madagascar	1	-	-	-
Martinique	1	-	-	-
Mexico	1	-	-	-
Namibia	6	4	1	66.7
Niger	15	5	2	33.3
Nigeria	51	27	9	52.9
Saudi Arabia	3	-	-	-
Senegal	1	-	-	-
Somalia	1	-	-	-
South Africa	2	2	1	100.0
Sudan	15	10	2	66.7
United Kingdom	5	3	1	60.0
**Total**	**318**	**148**	**50**	**46.5**

The table shows the different countries of sampling of HBV/E sequences, the number of sequences sampled from each one of the countries, the number of samples that clustered and the corresponding number of sequences that clustered for each country as a percentage.

After the classification of countries into geographic regions, the distribution of HBV/E sequences per region was as follows: West Africa: 216 (67.9%), Central Africa: 51 (16%), East Africa: 18 (5.7%), Europe: 11 (3.5%), Americas: 8 (2.5%), Southern Africa: 8 (2.5%), North Africa/Middle East: 5 (1.6%) and Asia: 1 (0.3%). The sequences sampled from these geographical regions showed no statistically significant differences in the nucleotide diversity ranging between 0.7% and 2% (S2A and S2B Table) with West and Central Africa having the highest diversity.

**Fig 2:**
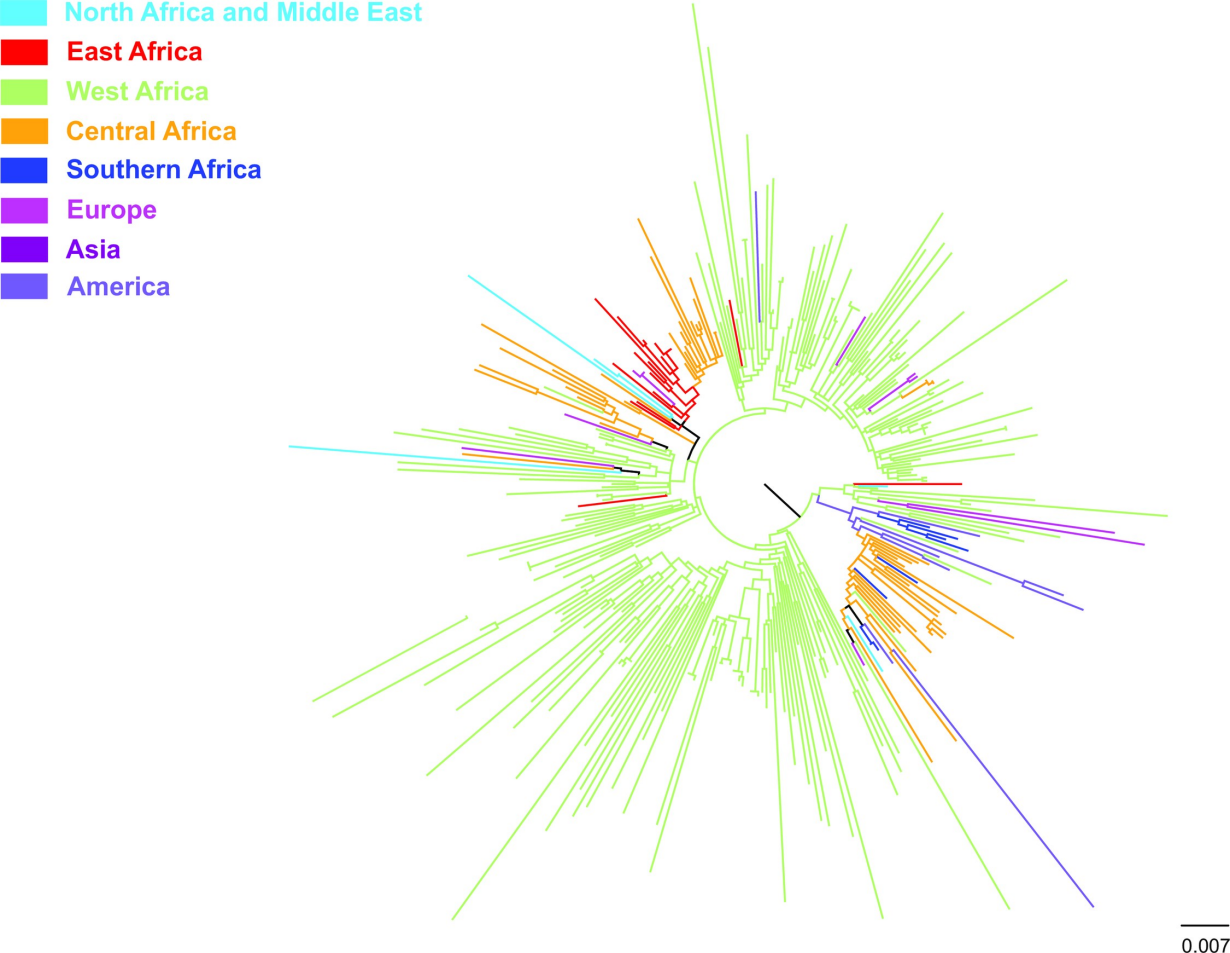
Global dispersal of HBV/E strains between the geographical regions. A midpoint rooted phylogeographic tree estimated by RAxML v8.0.20. HBV/E sequences (N = 318) used in the analysis are categorized according to the geographic region of sampling.

Phylogeographic analysis of the HBV/E sequences grouped in geographic regions revealed the existence of local dispersal in Africa (Fig 2). In addition, sequences from West Africa were located close to the root of the ML tree indicating that the HBV/E epidemic probably originated in West Africa and expanded to other geographical regions, within and outside of Africa (Fig 2). There are also some indications that the European strains originated primarily from West Africa whereas Western Central Africa was the source of the majority of viral strains dispersed to the Americas (Fig 2).

Country-wise phylogeographic analysis suggests that 46.5% (148) of the total number of HBV/E sequences (N = 318) were found within 50 monophyletic clusters (Table 1). The analysis showed that HBV/E sequences form regional clusters at different percentages according to their geographic origin (Table 1). Specifically, all the sequences sampled from Democratic Republic of the Congo form a single monophyletic cluster. The same pattern was observed for Colombia, Egypt and South Africa. High levels of local dispersal, where > 50% of sequences showed monophyletic clustering, were found for Cameroon, Ghana, Liberia, Namibia, Nigeria, Sudan, and United Kingdom (Table 1).

A number of sequences from Guinea and Nigeria formed 14 and 9 monophyletic clusters, respectively whereas for Belgium, Cameroon, Central African Republic, Colombia, Democratic Republic of Congo, Egypt, Ghana, Liberia, Namibia, Niger, South Africa, Sudan and United Kingdom, a limited number of clusters were detected ranging from one to six (Table 1). The sequences sampled from two semi-isolated rural communities in North and Central Nigeria clustered in a single, separate clade indicative of localized intra-country dispersal. The <50% monophyletic clustering of sequences from Belgium, Central African Republic, Guinea and Niger revealed the lowest regional dispersal. None of the sequences from Angola, Argentina, Benin, Burkina Faso, Cape Verde, Cuba, Ethiopia, Japan, Madagascar, Martinique, Mexico, Saudi Arabia, Senegal and Somalia formed monophyletic clusters (Table 1).

## Discussion

Wide-range full-genome phylogenetic and phylogeographic analyses of the dispersal patterns of HBV/E were performed. As HBV/E is predominantly found in West Africa, there was an over-representation of some countries/geographical regions, probably introducing a sampling bias that cannot be avoided. Nonetheless, despite the limitations under these assumptions, the full-length HBV/E sequences analyzed showed a conspicuous low genetic diversity of 1.95% similar to earlier studies that reported an intragenotypic nucleotide divergence of 1.73% [15, 18, 26]. The low nucleotide diversity suggests its relative recent introduction into the population [26]. This coincides with reports that concluded that the recent origin and wide distribution of HBV/E in the West African crescent suggests a rapid population expansion of HBV/E infections [53].

The present analyses of the limited number of sequences available in the databases, suggest that HBV/E sequences found in the European region and in the Americas were disseminated mostly from West African region. Considering HBV/E is only intermittently found in the Americas and rarely found outside Africa except in individuals of African descent, [20] this analysis is based on a small number of sequences thus limiting our ability to reach firm conclusions or make a strong statement.

Various times from the most recent common ancestor (t_MRCA_) of HBV/E have been calculated using Bayesian inference, with a median time from t_MRCA_ of 130 years [28] whereas in Nigeria, a more recent t_MRCA_ was estimated to be year 1948 (95% HPD: 1924–1966), with an increase of HBV/E-infected population over the last ~40 to 50 years [53]. These times differ from the estimated t_MRCA_ of 6,000 years [29]. However, as previously suggested HBV/E may have existed in indigenous African populations and recently re-introduced [15]. HBV/E has previously been isolated in individuals from Colombia [54], India [55], Pygmies [56] and the Khoi San (Kramvis, unpublished data), with no history of travel to or from Africa. Nonetheless, resolution of the variance of the estimated age of HBV/E will be difficult without the accurate determination of the nucleotide substitution rate of HBV [13]. In contrast, the presence of subgenotype A1 in Brazil and Haiti [27, 57], coincides with the present dominance of this subgenotype in southeast Africa, which was the source of the ~ 400, 000 captives taken to south and Central America in the middle of the 19^th^ century. The fact that HBV/E did not cause an epidemic in the Americas could be because of the absence of HBV/E infection in the founding population of slaves or the limited secondary onward transmission within this population.

**Fig 3:**
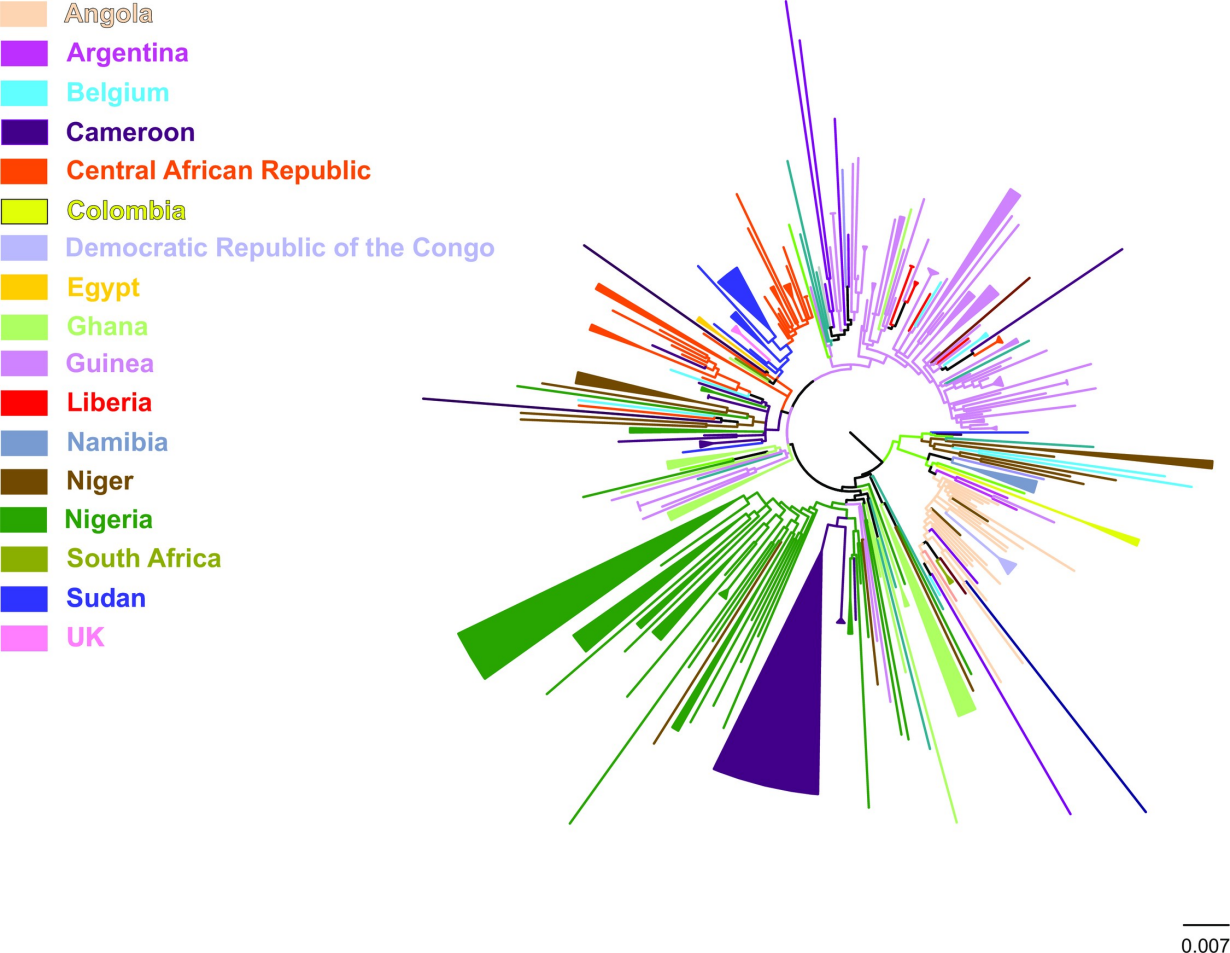
Country-wise dispersal of HBV/E strains. A midpoint rooted phylogeographic tree estimated by RAxML v8.0.20. HBV/E sequences (N = 318) used in the analysis are categorized according to the country of sampling. Monophyletic clusters are indicated as solid triangles.

The observed pattern of regional dispersal for sequences sampled from Nigeria and Guinea (Fig 3) suggests limited population movements associated with cross-border transmissions. In addition, the sequences sampled from Nigeria clustering in a separate clade supports the limited cross border transmission. The rapid spread of HBV/E within a short period that was observed in large parts of Africa can be associated with a sudden change in the route of transmission. It is plausible that a sudden change in the route of transmission [20] such contaminated vaccine preparations [27] may be responsible for the spread. Furthermore, numerous mass injection campaigns against small pox, yaws [27, 58, 59] and sleeping sickness [60], using multiple injections with same needles, were undertaken in the West African region. In addition, socio-cultural practices like facial or body scarification, traditional birth attendance and shaving by local barbers using unsterilized sharp instruments are alternative routes of transmission of blood-borne pathogens [61, 62]. A study conducted in Egypt linked the transmission of HCV to unsafe mass injection campaign against schistosomiasis until the 1980’s [63]. Therefore, because HBV is more transmissible than HCV, [64] it may partly explain the rapid spread of the HBV/E in West Africa [63]. The big puzzle to be solved is the reason HBV/E rapidly spread in West Africa and predominated over genotype A, which was dispersed from Africa by slave trade to the Americas [14, 65].

Perinatal transmission is possibly another mode of HBV transmission that might have led to the rapid spread of HBV/E in sub-Saharan Africa. HBeAg easily crosses the placenta to infants born to HBeAg-positive mothers infected with HBV/E (vertical) [66]. This can lead to HBe/HBcAg tolerance *in utero* and perinatally [37] thus there is a high probability of chronic carrier status later in life [14, 26, 34, 35, 37, 67, 68]. In addition, community based transmission (horizontal) caused by children coming to contact with open wounds including behavioral factors (biting of fingernails and scratching the back of the carriers), sharing of bath towel and dental cleaning materials [69, 70] is another mode of transmission. Extensive studies have been done to further identify the factors that influence perinatal transmission but with limited focus on West Africa. Although perinatal HBV transmission may explain, in part, the explosive spread of virtually identical viruses within a community, it is critical to understand whether it also explains the similarity of viruses across the vast expanses of the HBV/E crescent.

A study, conducted by Jayaraman and colleagues, linked the rapid spread of HBV and HIV infection in sub-Saharan Africa to the risky practices including blood transfusion and socio-cultural practices [64]. Most of the sequences sampled from the different geographical regions were obtained from asymptomatic carriers, blood donors or ESLD patients infected with HBV/E. The progression of chronic HBV to cirrhosis, end stage liver disease (ESLD) and hepatocellular carcinoma (HCC) is more rapid in HIV-positive individuals than those with HBV alone [71]. The onset of the HIV epidemic in the 1950’s might have played a role in the explosive transmission and dispersal of HBV/E in West Africa [72] with a high frequency of HBV/HIV co-infection [73].

## Conclusion

Taken together, our findings suggest considerable differences in the pattern of HBV/E regional dispersal, with the HBV/E epidemic probably originating in the West Africa and expanding to other regions, within and outside Africa. The observed strong patterns of regional and localized dispersal suggest that the population movements associated with cross-border transmissions were limited and this could be explained by the late introduction of HBV/E into the population as well as a sudden change in the route of transmission such as extensive use of unsafe needles in mass immunization campaigns and socio-cultural practices. In addition, the onset of the HIV epidemic in the 1950’s might have played a role in the explosive transmission and dispersal of HBV/E in West Africa, where HBV/HIV co-infection rate is high.

## Supporting information

S1 Table A. Overall intragroup sequence divergence. This is the table showing the overall genotype E sequence divergence for sequences sampled from all the 28 countries. B. Intragroup sequence divergence within the countries. This is the table showing the diversity of genotype E sequences within the different countries in which the sequences were sampled from. C. Intergroup sequence divergence between the countries. This is the table showing the diversity of genotype E sequences between the different countries in which the sequences were sampled from.
(XLSX)

S2 Table A. Intragroup sequence divergence within the different geographical regions. This is the table showing the diversity of genotype E sequences within the different geographical regions in which the sequences were sampled from. B. Intergroup sequence divergence between the different geographical regions. This is the table showing the diversity of genotype E sequences between the different geographical regions in which the sequences were sampled from.
(XLSX)

S3 Table (XLSX)
